# Surgical Outcomes of Hidradenitis Suppurativa Excision: Factors Impacting Wound Healing Time

**DOI:** 10.1111/wrr.70190

**Published:** 2026-08-08

**Authors:** Christina Huang, Scott Koeneman, Sherry Yang, Sami Tannouri

**Affiliations:** ^1^ Sidney Kimmel Medical College Thomas Jefferson University Philadelphia Pennsylvania USA; ^2^ Department of Pharmacology, Physiology, & Cancer Biology Thomas Jefferson University Philadelphia Pennsylvania USA; ^3^ Department of Dermatology and Cutaneous Biology Thomas Jefferson University Philadelphia Pennsylvania USA; ^4^ Department of Surgery Thomas Jefferson University Philadelphia Pennsylvania USA

**Keywords:** dehiscence, hidradenitis suppurativa, perioperative biologics, wound healing

## Abstract

Hidradenitis suppurativa (HS) can be treated surgically to excise recurrent and recalcitrant regions of disease activity. However, surgical outcomes are highly variable depending on closure method, concomitant medical management and area of excision. In this study, we aim to characterise the healing courses from HS excision in a case series. To reduce operator variability, we opted to conduct a case series from a single surgeon. Surgeries conducted from June 2024–December 2025 by surgeon S.T. were included, and the postoperative course was followed until February 2026. Surgical details and postoperative course for 34 sites of HS across 20 patients were analysed. For operations undergoing primary closure (14/34), median wound healing time was 131 days, while sites healing by secondary intention (20/34) had a median wound healing time of 161 days. Four out of seven surgically closed sites on patients undergoing tumour necrosis factor alpha (TNF‐*α*) inhibitor therapy experienced dehiscence, while six out of ten surgically closed sites while the patient was on interleukin‐17 (IL‐17) inhibition experienced dehiscence. Surgical excision of HS with either primary or secondary closure had a median wound healing time of 138 days. Primary closure of excisions, even with mobilisation of flaps to reduce tension, results in high rates of dehiscence, especially for patients on adjuvant biologic therapy. Our limited case series observed high rates of dehiscence amongst patients receiving either IL‐17 inhibitors or TNF‐*α* inhibitors. To our knowledge, this is the first study comparing wound healing between patients on TNF‐*α* inhibitors versus IL‐17 inhibitors versus no biologic therapy. HS patients considering surgery should be counselled on these possible risks and expected wound healing times for primary versus secondary intention healing.

**Abbreviations**
HS: hidradenitis Suppurativa
IL‐17: interleukin 17
TNF‐*α*
: tumour necrosis factor alpha

## 1 Introduction

Hidradenitis suppurativa (HS) is a chronic inflammatory disease characterised by recurrent painful nodules, abscesses and tracts favouring intertriginous regions [1]. Medical management alone is often insufficient in controlling advanced disease and surgical intervention may be necessary to target particularly recurrent regions of inflammation or refractory disease [2]. Surgical options include incision and drainage, deroofing and radical/wide local excision, wherein the entire region of affected skin is excised down to the uninvolved subcutaneous fat [3]. Notable procedure‐specific risks associated with surgical excision include wound infections (25%), wound dehiscence (30%), hyper‐granulation (25%), disease recurrence (30%), chronic pain, abnormal scarring and scar contractures [4].

Previous studies have suggested that the method of repair following wide excision may significantly impact the likelihood of post‐surgical complications. Delayed closure by secondary intention may be associated with reduced adverse effects during the healing window, despite the need for more active wound care [5]. Other factors that may delay wound healing after HS excision include anatomical area and presence of comorbid inflammatory conditions [6]. Postoperative care also has an important impact on wound trajectory, and guidelines on optimal postoperative wound management—from use of advanced wound therapy to dressing materials—for HS excisions have yet to be established [7]. However, elucidating the impact of these surgical factors proves challenging given inconsistently reported confounders across datasets, including HS severity, concurrent medical treatments and differing techniques between surgeons [8].

Furthermore, results of HS excision in the setting of concurrent biologic treatment, including with the interleukin‐17 (IL‐17) inhibitor class, have not been fully explored. Perioperative continuation of adalimumab, a tumour necrosis factor alpha (TNF‐*α*) inhibitor, was shown to improve outcomes after surgery for patients with moderate to severe HS [9]. However, the study was limited to surgeries with healing by secondary intention and therefore did not include operations with sutured repairs.

To characterise surgical outcomes while limiting operator variability, we conducted a study of a single‐surgeon's case series of HS excisions to investigate factors that may affect wound healing, including biologic treatment and site of surgery.

## 2 Methods

**FIGURE 1:**
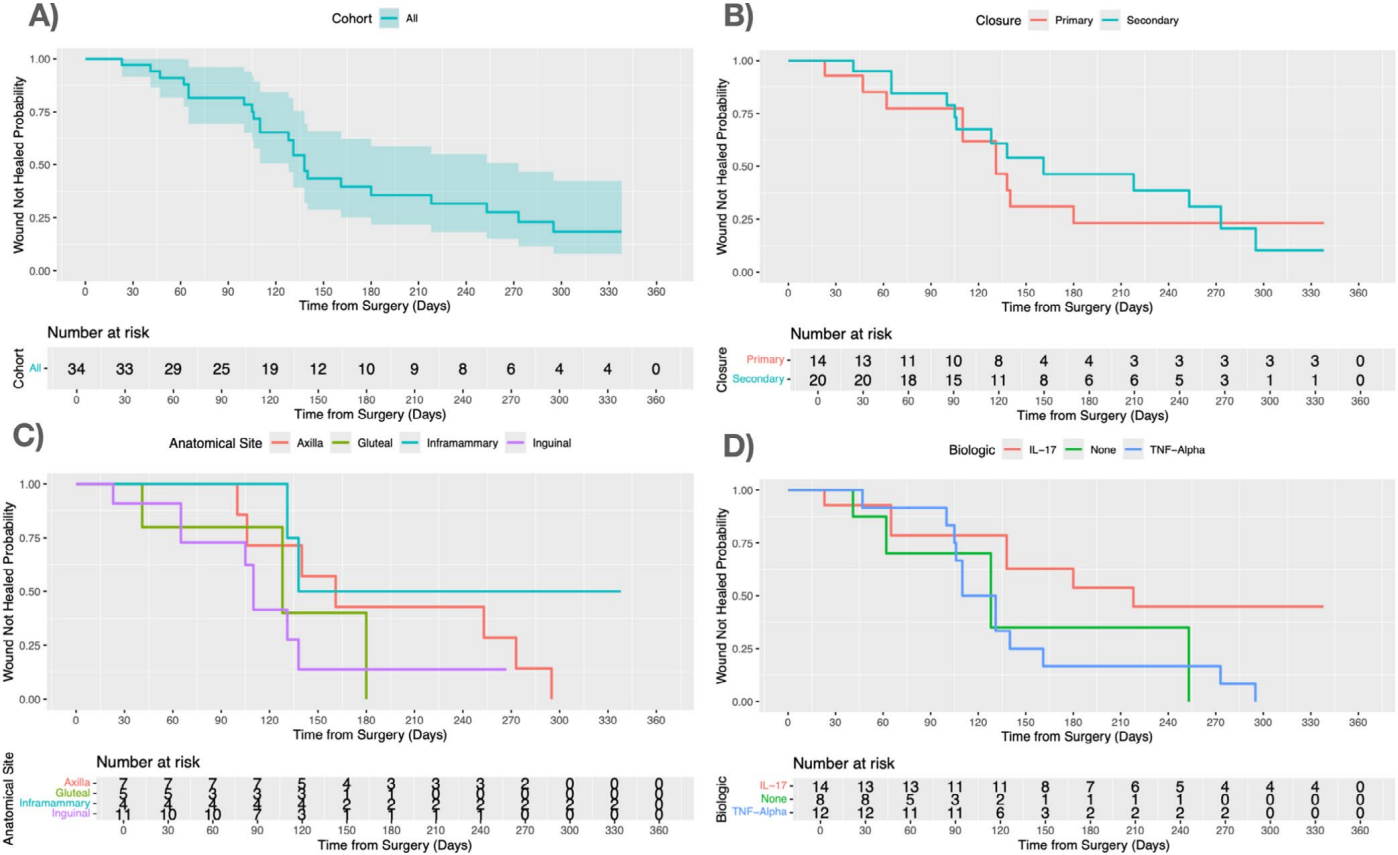
Kaplan–Meier estimation on percentage of wounds estimated to be healed at certain intervals over time for (A) all patients, (B) by closure method, (C) by anatomical site and (D) by biologic treatment.

Medical records of patients who underwent wide surgical excision of HS lesions from June 2024—December 2025 with author S.T. were reviewed. In total, 20 patients (average age 46, 70% female) underwent excisions for 34 total distinct sites of HS. Data collected included details of the surgery, postoperative course and patient characteristics. Survival analysis was performed to analyse time until complete wound healing, defined as full re‐epithelialisation of the surgical site, and a subsequent Kaplan–Meier estimate of probability of freedom from wound healing across time was generated and used to estimate median wound healing time in subsets of patients (Figure 1). Separate instances of HS were treated as statistically independent events, even if occurring in the same patient, for simplicity of the survival analysis given sample size constraints. Patients who were expired, lost to follow‐up, or not fully healed by the time of analysis (December 2025) were censored. Partial closures (wherein the lateral edges of the wound were sutured with a central portion deliberately left open) were grouped with secondary intent sites for the purposes of survival analysis; any instances of dehiscence occurring within the sutured portions were recorded for outcomes.

## 3 Results

The key results from the case series are summarised in Table 1.

**TABLE 1:** Summary of hidradenitis suppurativa excisions, biologic medications, healing course and patient characteristics.

Patient #	Age	Gender	Hurley stage	Biologic therapy	HS excision site	Dimensions of excision	Method of closure	Days to fully healed	Incidence of dehiscence	Advanced wound therapy	Other adverse events	Tobacco use	BMI
1	30	F	III	Infliximab	Right inguinal	3 × 0.5 cm	Primary closure—simple interrupted sutures	131	No dehiscence			Never	38.9
1	30	F	III	Infliximab	Left inframammary	3 × 0.5 cm	Primary closure—simple interrupted sutures	131	No dehiscence				
1	30	F	III	Infliximab	Right inguinal	7 × 3 cm	Mobilised skin flap—primary closure with vertical mattress sutures	110	Yes		Hyper‐granulation		
1	30	F	III	Infliximab	Left inguinal	11 × 5	Primary closure—simple interrupted sutures	110	Yes		Hyper‐granulation		
2	46	F	II	Secukinumab	Left inframammary	4 × 1 cm	Primary closure—vertical mattress sutures	138	Yes			Never	40.6
2	46	F	II	Secukinumab	Right inguinal	15 × 14 cm	Mobilised skin flap—partial primary closure with vertical mattress sutures. Wound left open from midpoint to heal with secondary intention.	138	No dehiscence				
3	57	F	III	Infliximab	Left axilla	18 × 15 cm	Open	100	N/A–open	Wound VAC		Former smoker, 9 pack years	41.2
4	27	F	II–III	Infliximab	Left axilla	11 × 2 × 2 (depth) cm	Oasis graft in wound bed with mobilised flap closure	140	Yes			Never	
4	27	F	II‐III	Infliximab	Right axilla	12 × 4 × 3 cm	Oasis graft left open with wound vac	295	N/A–open	Wound VAC	Hyper‐granulation		44.5
5	59	F	III	Bimekizumab	Right thigh	29 × 15 × 5 cm	Open	157^a^	N/A–open		Found to have SCC in site of resected tissue; wound infection with chronic osteomyelitis treated with vancomycin and piperacillin/tazobactam post‐op	Active, 21 pack years	21.6
6	26	M	III	Adalimumab	Left axilla	14 × 9 × 3 cm	Open	273	N/A–open	Wound VAC		Never	29.7
7	37	M	III	Bimekizumab	Left medial thigh	18 × 10 cm	Open	206	N/A–open		Hyper‐granulation	Never	32.5
8	54	F	III	Infliximab	Left inguinal	25 × 10 × 4 cm	Open, packed w/betadine‐soaked Kerlix	105	N/A–open		Hyper‐granulation; acute sepsis and cellulitis pre‐op, on vancomycin and piperacillin/tazobactam perioperatively	Former, 30 pack years	40.7
9	30	F	III	Secukinumab	Left gluteus	12 × 3 cm	Mobilised skin flap—primary closure with vertical mattress sutures	180	Yes			Never	31.7
10	48	F	III	Bimekizumab	Right inguinal ^a^Note: Unroofing	^a^Not specified (unroofing, not excision)	Open	267^a^	N/A–open			Former, 10 pack years	31.47
10	48	F	III	Secukinumab	Right inframammary	16 × 7 cm	Primary closure—vertical mattress sutures	338^a^	Yes	Wound VAC			
10	48	F	III	Secukinumab	Abdominal wall left lower quadrant	15 × 16 cm	Panniculectomy Style flaps—running suture for fascia, primary closure of skin—vertical mattress sutures	338^a^	Yes	Wound VAC after dehiscence			
10	48	F	III	Secukinumab	Left inframammary	9 × 3 cm	Primary closure—vertical mattress sutures	338^a^	Yes	Wound VAC			
10	48	F	III	Bimekizumab	Complex abdominal wound closure (area of previous HS excision)	15 × 4 cm	Partial closure—simple interrupted (open medially)	84^a^	Yes	Wound VAC			
11	43	M	III	Bimekizumab	Left inguinal	18 × 6 cm	Partial closure—simple interrupted (open medially)	65	N/A–open			Never	31.8
11	43	M	III	Bimekizumab	Left lower inguinal	6 × 2 cm	Primary closure—simple interrupted sutures	23	No dehiscence				
11	43	M	III	Bimekizumab	Right inguinal	21 × 6 cm	Partial closure—simple interrupted (open medially)	65	N/A–open				
12	34	F	III	None	Left axilla	12 × 9 cm	Open	253	N/A–open		Hyper‐granulation	Former, 4 pack years	35.6
13	66	M	III	None	Perianal	3 × 2 cm	Partial closure—simple interrupted (open medially)	41	None			Former, 10 pack years	29.8
13	66	M	III	None	Left gluteus	8 × 4 cm	Partial closure—simple interrupted (open medially)	128	No dehiscence				
13	66	M	III	None	Left medial thigh	4 × 15 cm	Mobilised skin flap—primary closure with vertical mattress sutures	62	No dehiscence	Wound VAC			
14	43	F	II	Adalimumab	Right axilla	7 × 8 cm	Mobilised Z‐plasty skin flap—primary closure with vertical mattress sutures. Small medial portion left open due to tension	161	Yes			No	31.5
15	59	M	III	Infliximab	Abdominal wall	44 × 9 cm	Complex closure with plastic surgery: flaps mobilised with primary closure	47	No dehiscence			No	27.8
16	23	F	III	Adalimumab	L axilla	10 × 14 cm	Open	106	N/A–open			No	32.4
17	53	F	III	None	R inguinal	9 × 5 cm	Mobilised skin flap—partial closure with simple sutures	111^a^	Yes		Irritation from sutures	Never	31.8
18	64	M	III	Bimekizumab	R gluteus	29 × 25 cm	Open	125^a^	N/A–open			Former (4.8 pack years)	24.4
19	52	F	III	None	R medial thigh	25 × 9 cm	Partial closure—vertical mattress sutures (open medially)	46^a^	Yes			Yes (5.2 pack years)	43
19	52	F	III	None	L gluteus	10 × 5 cm	Primary closure with vertical mattress sutures	46^a^	Yes				
20	53	F	III	None	R inguinal	15 × 9 cm	Mobilised skin flap—partial primary closure with simple interrupted sutures	69^a^	No		Hyper‐granulation	Former (43.5 pack years)	20.1

^a^ Wound healing time censored in survival analysis due to patient expiration, loss of follow‐up or not fully healed by date of analysis.

For operations undergoing primary closure (14/34), median wound healing time estimated to be 131 days, while sites healing by secondary intention (20/34) had an estimated median wound healing time of 161 days. Wounds left to heal by secondary intention demonstrated higher mean surface area compared with primary closures (173 cm^2^ vs. 75 cm^2^, *p* = 0.02 Wilcoxon rank‐sum). Postoperative wound care for wounds healing by secondary intention was deliberately selected to be reproducible, safe, effective and with accessible wound care supplies as patients had varying access to home wound care specialists and supplies. Wound care instructions were given such that the patients were allowed to allow running water over their wounds (e.g., showering or wound irrigation) but without submerging the wounds during the healing period. The wounds were to be pat dried or air dried slowly. No topical agents were advised to be used. The wound bases were covered with non‐adherent petroleum based gauze to prevent sticking, painful dressing changes and bleeding. For the first 1–2 weeks iodine based petroleum gauze (Xeroform) was utilised, with the advice then to transition to non‐iodine‐based petroleum gauze dressings (Adaptic). A dry dressing was then placed over the non‐adherent dressings such as 4 × 4 gauze and/or Abdominal Pads. Overall, the use of tape and other adhesive materials was generally discouraged. Instead, garments such as surgical bras, supportive underwear, ace wraps or gauze webbing or rolls were used to secure the gauze layers over the wounds. For the first month, dressings were to be changed whenever saturated, up to three times per day, and to decrease the frequency of dressing change down to daily after the exudative period of wound healing had finished. Postoperative checks at the surgeon's office were performed weekly for the first month, then transitioned to every 2–3 weeks depending on the speed and progress of the wound healing until fully closed. Wound measurements and photos were tracked during outpatient visits. Patients had access to visiting wound care nurses or techs up to three times per week depending on the individual patient's needs and insurance provisions. Wound care strategies were deliberately kept simple to avoid confusion and high variation due to the different recommendations and experience of various wound care home agencies.

The use of vacuum‐assisted closure devices was limited to specific cases with particularly problematic wound healing trajectories. Vacuum‐assisted closure devices were used in four secondary intent healing sites, three after primary closures and one primary closure site post‐wound dehiscence.

### 3.1 Incidence of Wound Dehiscence for Closures

Among the 34 operations, there were 14 primary (8 linear, 6 flap repairs), 10 partial (6 linear, 4 flaps) and 10 secondary intent closures. Primary closure was selected in wounds where the width to length ratio along Langer's lines was 3:1 or higher yielding the possibility of a low‐tension closure. Surgical subcutaneous drains were also used at the surgeon's discretion. Wound dehiscence occurred in 13/24 (54%) of sutured repairs (4 partial, 9 primary) and across all anatomic sites, with a median onset of 17 days postoperatively. Six out of ten flap closures exhibited dehiscence despite adequate skin flap mobilisation to decrease incisional tension and the use of subcutaneous drains. There was no significant difference in mean excised surface area between wound sites that experienced dehiscence versus those that remained intact (74 cm^2^ vs. 99 cm^2^, *p* = 0.97 Wilcoxon rank‐sum).

### 3.2 Anatomical Site

The median wound healing times for axillary (*n* = 7), gluteal/perianal (*n* = 5), inframammary (*n* = 4) and inguinal (*n* = 11) sites of HS excision were 161, 128, 238 and 110 days respectively. The median excision area at these sites was 108, 36, 41 and 66 cm^2^. Factors such as greater mobility and increased pilosebaceous units in axillary sites could also contribute to variation in wound healing (6). Median healing time could not be calculated by survival analysis for abdominal and thigh sites as only zero or one wounds were healed at the time of analysis. Healing time differed across anatomical sites, although small sample size made the analysis inconclusive.

### 3.3 Effect of Biologics

Twenty‐six out of 33 (76%) excisions occurred in patients on biologic medications for HS, all of which were continued perioperatively without changes in dosing regimen. Twelve were on TNF‐*α* inhibitors, 14 were on IL‐17 inhibitors and 8 were not on any biologic agent. Median wound healing times for these surgical sites were 128, 218 and 128 days, respectively. For primary and partially sutured closures, dehiscence occurred in 4/7 sites in patients on anti‐TNF‐*α*, 6/10 on anti‐IL‐17 therapy and 3/7 on no biologics. Subjectively, patients on immunologic therapy who developed wound dehiscence were noted to have minimal fibrin deposition within the dehisced sites upon post‐surgical follow‐up, suggesting disruptions in the normal wound healing pathway.

## 4 Discussion

This case series demonstrates the estimated healing course for HS patients undergoing wide surgical excision at a single academic referral centre. Results of this study can help guide surgical decision‐making and expectations for patients. In the clinical setting, patients may opt for primary closure if they cannot care for an open wound for an extended period of time but should be counselled on the relatively high rates for dehiscence. Furthermore, for secondary wound closures, no established guidelines exist regarding optimal post‐surgical HS wound care, but important principles of reducing bacterial load, having appropriate moisture levels and promoting granulation tissue [7]—as well as patient access to wound care supplies and services—must be considered for wound care for patients healing with secondary intention [7].

The effect of biologics on primary closure healing course is not well established. To our knowledge, this is the first study examining differences in wound healing between patients on TNF‐*α* inhibitors versus IL‐17 inhibitors. Furthermore, this study includes primary closure outcomes for patients on TNF‐*α* inhibitors. In this study, median wound healing time while on IL‐17 inhibitor therapy was 218 days, compared to 128 days for patients on TNF‐*α* inhibitor therapy and 128 days for patients on no biologic therapy. Multiple factors could have contributed to this difference, as wound size, patient comorbidities, wound site and other variables were not controlled for. However, further studies into the effect of IL‐17 inhibition therapy on wound healing times are warranted, considering the 90‐day difference on median wound healing time in our cohort. Given that IL‐17 activates multiple pathways during the proliferative phase of wound healing, inhibition of this cytokine may negatively impact primary wound closure [10]. Notably, IL‐17 contributes to keratinocyte migration and proliferation and fibroblast activation which are crucial for re‐epithelisation and extracellular matrix deposition respectively [11]. Furthermore, in vitro studies have found that TNF‐*α* inhibitors induce a wound healing profile via macrophage and matrix metalloproteinase expression, which may contribute to the discrepancy in healing times between biologics in our study cohort [12].

Study limitations include small sample size, assumed statistical independence of HS excisions occurring within a single patient, and lack of generalisability given the single centre, single operator design as the trade‐off for having increased operator consistency. In addition, other important factors that may have affected wound healing, such as postoperative wound dressings, advanced wound therapy, age and comorbid conditions, were not further investigated for their impact on wound healing time.

## 5 Conclusions

Surgical excision of HS with either primary closure or secondary intent healing at our centre had a median wound healing time of 4 to 5 months. Primary closure of excisions, even with mobilisation of flaps, resulted in high rates of dehiscence. Further investigation to characterise and identify factors that impact wound healing, especially in regard to concurrent biologic therapy, will improve surgical counselling and perioperative optimisation in the future.

## Author Contributions

Christina Huang contributed to writing (original draft) and data curation for this study. Scott Koeneman contributed to formal analysis and writing (review and editing). Sherry Yang contributed to conceptualization of this project, methodology, and writing (review and editing). Sami Tannouri contributed to the conceptualization of this project, data curation, methodology, and writing (review and editing).

## Funding

The authors have nothing to report.

## Consent

This study was deemed exempt from Institutional Review Board approval at Thomas Jefferson University.

## Conflicts of Interest

The authors declare no conflicts of interest.

## Data Availability

The data that support the findings of this study are available on request from the corresponding author. The data are not publicly available due to privacy or ethical restrictions.
